# Reduced Retinal Degeneration in an Oxidative Stress Organ Culture Model through an iNOS-Inhibitor

**DOI:** 10.3390/biology10050383

**Published:** 2021-04-28

**Authors:** Ana M. Mueller-Buehl, Teresa Tsai, José Hurst, Carsten Theiss, Laura Peters, Lisa Hofmann, Fenja Herms, Sandra Kuehn, Sven Schnichels, Stephanie C. Joachim

**Affiliations:** 1 Experimental Eye Research Institute, University Eye Hospital, Ruhr-University Bochum, In der Schornau 23–25, 44892 Bochum, Germany; Ana.Mueller-Buehl@rub.de (A.M.M.-B.); teresa.tsai@rub.de (T.T.); Laura.Peters@rub.de (L.P.); lisahof_91@yahoo.de (L.H.); fenja.herms@gmx.de (F.H.); Sandra.Kuehn@rub.de (S.K.); 2 Center for Ophthalmology Tübingen, University Eye Hospital Tübingen, 72076 Tübingen, Germany; jose.hurst@med.uni-tuebingen.de; 3 Department of Cytology, Institute of Anatomy, Ruhr-University Bochum, 44801 Bochum, Germany; Carsten.Theiss@rub.de; 4 Clinic for Small Mammals, Reptiles and Birds, University of Veterinary Medicine, 30559 Hannover, Germany

**Keywords:** oxidative stress, iNOS-inhibitor, porcine organ culture model, apoptosis, autophagy

## Abstract

**Simple Summary:**

There is an urgent need to develop new therapeutic approaches for diseases of the retina, like glaucoma. In their pathogenesis, oxidative stress and the corresponding defense reactions play an important role. In porcine retinal organ cultures, hydrogen peroxide can be used to simulate oxidative stress. In the present study, we investigated whether the treatment with an inducible nitric oxide synthase inhibitor protects retinal cells from oxidative stress. Therefore, porcine retinal explants were damaged with hydrogen peroxide and treated with the nitric oxide synthase inhibitor. Analyzes of the retina at four and eight days showed that a inhibitor was able to prevent degeneration in porcine retinas, since retinal ganglion cells were protected to some extent. Moreover, in the later course, there was also protection of other retinal cells (bipolar cells). Hence, this inhibitor seems to be a promising treatment option for retinal diseases.

**Abstract:**

In retinal organ cultures, H_2_O_2_ can be used to simulate oxidative stress, which plays a role in the development of several retinal diseases including glaucoma. We investigated whether processes underlying oxidative stress can be prevented in retinal organ cultures by an inducible nitric oxide synthase (iNOS)-inhibitor. To this end, porcine retinal explants were cultivated for four and eight days. Oxidative stress was induced via 300 µM H_2_O_2_ on day one for three hours. Treatment with the iNOS-inhibitor 1400 W was applied simultaneously, remaining for 72 h. Retinal ganglion cells (RGC), bipolar and amacrine cells, apoptosis, autophagy, and hypoxia were evaluated immunohistologically and by RT-qPCR. Additionally, RGC morphology was analyzed via transmission electron microscopy. H_2_O_2_-induced RGCs loss after four days was prevented by the iNOS-inhibitor. Additionally, electron microscopy revealed a preservation from oxidative stress in iNOS-inhibitor treated retinas at four and eight days. A late rescue of bipolar cells was seen in iNOS-inhibitor treated retinas after eight days. Hypoxic stress and apoptosis almost reached the control situation after iNOS-inhibitor treatment, especially after four days. In sum, the iNOS-inhibitor was able to prevent strong H_2_O-induced degeneration in porcine retinas. Hence, this inhibitor seems to be a promising treatment option for retinal diseases.

## 1. Introduction

Ex vivo organ cultures represent a unique research option as they combine both the advantages of animal models and those of cell cultures. Additionally, in ophthalmological research, which traditionally mainly uses laboratory animals or immortalized single cell cultures, organ cultures have increasingly come into focus. Among other things, an ex vivo corneal organ culture model, useful for wound healing studies, has been established [1] and the effectiveness of mesenchymal stem cells in reducing corneal scars has also been tested in an ex vivo organ culture model [2]. Additionally, research of retinal degeneration occurring in glaucoma, retinal ischemia, or age-related macular degeneration (AMD) is increasingly being carried out in ex vivo organ cultures [3,4]. In recent years, our research groups have adapted retinal organ culture models to investigate the pathomechanisms of retinal diseases and to test new therapeutic options [5,6,7,8]. These models are based on porcine retinas, which can be obtained as a waste product from the food industry. Morphology and physiology of the porcine retina is quite similar to the human retina, more so than those of commonly used (rodent) laboratory animals [5,9].

Both the structural and the functional integrity of the retina depend on its oxygen supply. As one of the most metabolically active tissues, the retina consumes oxygen much faster than all other tissues and is particularly prone to oxidative stress. Oxidative stress is defined as an imbalance between the production and elimination of reactive oxygen species (ROS) [10]. ROS arise under physiological conditions (e.g., during oxidative phosphorylation in mitochondria and in response to cytokines or exogenous stimuli) [10]. The resulting reactivity of ROS consists of the oxidation of lipids, proteins, and nucleic acids. In addition, it has also been shown that hormone-activated ROS can mediate the induction of hypoxia-inducible factor 1-alpha (HIF-1α), even under normal oxygen conditions [11]. By interacting with various signaling molecules, ROS influence cell proliferation, survival, differentiation, and metabolism as well as antioxidative and anti-inflammatory response [12]. ROS include various members like hydrogen peroxide (H_2_O_2_), singlet oxygen, and free radicals like nitric oxide (NO) [10].

As a signaling molecule, NO assumes various physiological functions such as initiation of vasodilation, neurotransmission, and—produced by macrophages—the host cell defense [13,14]. NO is synthesized from L-arginine via three isoforms of nitric oxygen synthase (NOS): the endothelial NOS (eNOS), neuronal NOS (nNOS), and inducible NOS (iNOS). The nNOS and iNOS isoform are present in the retina [15]. In contrast to eNOS and nNOS, which require calcium for their activation and continuously produce NO, iNOS is only expressed by an inflammatory stimulus. This stimulus leads to the activation of macrophages/microglia, which in turn express large amounts of iNOS and thus lead to high NO production [16]. Normally, ROS are rendered harmless by a good balance between pro- and antioxidative substances. When the formation of ROS exceeds the antioxidative capacity, oxidative stress arises. Oxidative stress occurs in numerous neurodegenerative diseases such as Parkinson’s or Alzheimer’s disease [17,18]. In glaucoma patients, increased intraocular pressure (IOP) can lead to oxidative stress, which in turn induces retinal ganglion cell (RGC) damage [19]. Furthermore, the overproduction of iNOS leads to large amounts of NO over a long period of time, so that NO is converted into NO_2_, nitrite, peroxynitrite, and free radicals. These in turn induce pathophysiological effects such as functional alterations involved in trabecular meshwork degeneration, retinal apoptosis, optic nerve degeneration, and posterior retinal degeneration lesions, which can be involved in glaucoma [20]. To prevent these eye diseases, oxidative stress inhibitors, more precisely inhibitors of the iNOS activity, seem to be an interesting therapeutic approach.

In recent years, iNOS inhibition has been primarily used in cancer research and therapy [21]. Lately, it has also made its way into the treatment of retinal diseases [22]. In a previous study, we investigated the neuroprotective effect of the iNOS-inhibitor 1400 W on CoCl_2_-induced hypoxic retinal damage and revealed a neuroprotective effect on RGCs and bipolar cells [5,23]. RGCs are the only projecting neurons in the retina transmitting visual information to the brain. RGCs suffer apoptotic cell death during development and in response to injury such as in glaucoma, retinal ischemia, and other optic neuropathies. The mechanisms of RGC death are still the subject of intense study, and various factors including oxidative stress have been suggested to be involved in RGC degeneration [24]. Further studies like the successful protection of RGCs by a topical treatment of glaucomatous mice with a P27 receptor antagonist underline the potential of therapeutic RGC protection for the treatment of numerous retinal diseases [25].

Therefore, in the present study, we examined the potentially protective effect of the iNOS-inhibitor 1400 W against oxidative damage in the retina, especially the degeneration of the RGCs. To this end, we applied H_2_O_2_, a strong oxidizing agent, to porcine retina explants [8]. The effects of the iNOS-inhibitor were determined by investigating markers for RGCs, bipolar, and amacrine cells as well as markers for apoptosis and autophagy. The results indicate that the iNOS-inhibitor has protective effects, especially on the RGCs of H_2_O_2_ damaged retinas, mainly based on the lowering of the apoptosis rate.

## 2. Materials and Methods

### 2.1. Subsection Preparation of Porcine Retinal Explants and Treatment Scheme of H_2_O_2_-Induced Degeneration

Porcine eyes were obtained from the local abattoir. Preparation of retinal explants was carried out as described previously [5,6,7,8]. Retinal explants were cultured on inserts (Merck Millipore; Burlington, MA, USA) in a 6-well plate with 1 mL Neurobasal-A-medium (Life Technologies, Carlsbad, CA, USA) supplemented with 0.8 mM·L-glutamine (Life Technologies), 2% B27 (Life Technologies), 1% N2 (Life Technologies), and 2% penicillin/streptomycin (Sigma-Aldrich, St. Louis, MO, USA).

Retinal explants were cultivated for four and eight days (n = 22/group for both points in time). In previous studies, we noted that our retina organ cultures should not be cultivated for more than eight days. Later on, the integrity of the retina and thus evaluable analyses were no longer possible [8].

In order to investigate the time course of the cell death mechanisms after iNOS-inhibition, an earlier point in time (four days) was also analyzed, since in previous studies, cell death could already be detected at an earlier point in time in the H_2_O_2_ degeneration model [6] and in a hypoxia degeneration model, protection through the iNOS-inhibitor could already be identified after four days [5].

**Figure 1:**
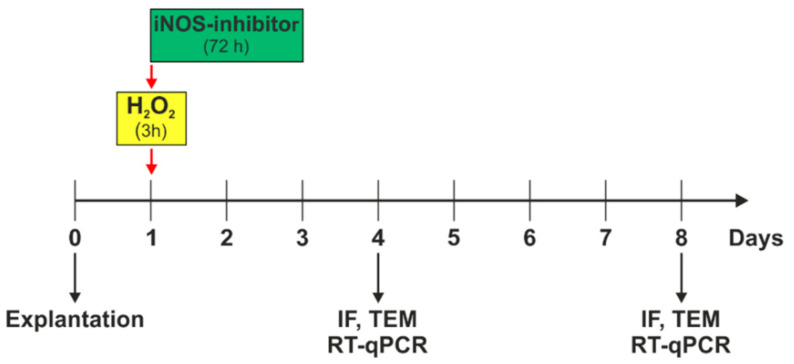
Experimental setup. Porcine retinas were cultivated for four and eight days. Oxidative stress was induced by 300 µM H_2_O_2_ on day one, for three hours. Simultaneously to the stressor, the iNOS-inhibitor treatment was applied and remained on retinal explants for 72 h. On day four, immunofluorescence (IF), transmission electron microscopy (TEM), and quantitative real-time PCR (RT-qPCR) analyses were performed. At day eight, IF, TEM, and RT-qPCR was evaluated.

On day one, degeneration via 300 µM H_2_O_2_ was induced for three hours. Simultaneously to the beginning of degeneration, treatment with 500 µM iNOS-inhibitor (1400 W, Merck Millipore) was started and remained for 72 h, until day four. Control retinal explants were cultivated without any further treatment during the whole cultivation period (Figure 1). A complete medium exchange was performed on days zero, one, two, and three. On days four, six, and seven, 50% of the medium was renewed.

For subsequent analyses, immunohistochemistry, RT-qPCR, and transmission electron microscopy, retinal samples were frozen.

### 2.2. Analyses of Immunohistological Stainings

For immunohistological analyses, retinal samples were fixed for 15 min using 4% paraformaldehyde. Then, samples were cryo-protected with 15% sucrose for 15 min, followed by 30% sucrose for 30 min, and frozen in liquid nitrogen. Retinal cross-sections (10 µm) were cut on a cryotome. Cross-sections for stainings of RBMPS, PKCα, calretinin, and HIF-1α were pre-incubated for one hour with blocking buffer consisting of 0.1–0.2% PBS/TritonX-100 (Merck Millipore) and 10–20% normal donkey or goat serum (Dianova, Hamburg, Germany). Primary antibodies were diluted in the same blocking buffer and incubated at room temperature overnight (Table 1). The next day, secondary antibodies were diluted in the same blocking buffer and incubated for one hour (Table 1). Cell nuclei were visualized with 4′,6′-Diamidin-2-Phenylindol (DAPI, Dianova). For antigen retrieval, cross-sections for stainings of cleaved caspase 3, Bax, and LC3B were additionally cooked in citrate buffer for 10 min.

For statistical evaluations (n = 9–10/group), six retinal slices per explant were used, in total, 24 masked images of each retina were counted for each staining. When specific markers were co-localized with DAPI, cells were counted as positive.

For measurement of the Tuj1^+^ area, images were transferred into grayscale (32-bit), as described previously [7,26]. Background was subtracted using a defined rolling ball radius (four days: 30.0; eight days: 50.0). A mean value for lower and upper thresholds were defined from thresholds of all images (four days: lower: 7.61; upper: 73.82; eight days: lower: 10.97, upper: 98.31).

### 2.3. Analyses of Quantitative Real-Time PCR (RT-qPCR)

Retinal samples (n = 4–8/group) were analyzed for the mRNA expression of specific markers (Table 2). mRNA was isolated and reverse transcribed using the MultiMACS mRNA and cDNA Synthesis Kit on the MultiMACS™ M96 Separator (Miltenyi Biotec, Bergisch Gladbach, Germany) according to the manufacturer’s instructions. RT-qPCR was performed with 40 cycles using the Universal SYBR Green Supermix (Biorad, Hercules, CA, USA). A total of 5 µL of cDNA (concentration 1 ng/µL) was used in a volume of 20 µL reaction mix. cDNA expression levels of investigated genes were normalized to the housekeeping genes β-ACTIN (ATCB, SI) and RLP4. The final primer concentration was 100 nM.

### 2.4. Transmission Electron Microscopy

Cultivated retinas (n = 2/group for each point in time) were used for transmission electron microscopy. Samples were fixed in 2.5% glutaraldehyde in 0.1 mol/L cacodylate buffer (pH 7.3). Post-fixation was performed in buffered 2% osmium tetroxide. Dehydrated retinas were embedded in araldite and ultra-thin sections were cut. Ultrastructural analyses were performed using a Philips EM 420 transmission electron microscope equipped with a digital CCD camera (Model 792 BioScan; Gatan, CA, USA).

### 2.5. Statistical Analyses

For immunohistological analyses, groups were compared by a one-way ANOVA followed by Tukey’s post-hoc test for unequal or equal groups (Statistica V12; Statsoft) and are presented as mean ± SEM. Cell counts are shown in cells/mm for each staining. In accordance, RT-qPCR data were analyzed using ANOVA, followed by Tukey’s post-hoc test to analyze the differences between groups (GraphPad Prism 8) and are presented as mean ± SD. *p*-values < 0.05 were considered as statistically significant with * *p* < 0.05, ** *p* < 0.01, and *** *p* < 0.001.

## 3. Results

### 3.1. Early Reduction of Hypoxic Stress Marker HIF-1α after iNOS-Inhibitor Treatment

**Figure 2:**
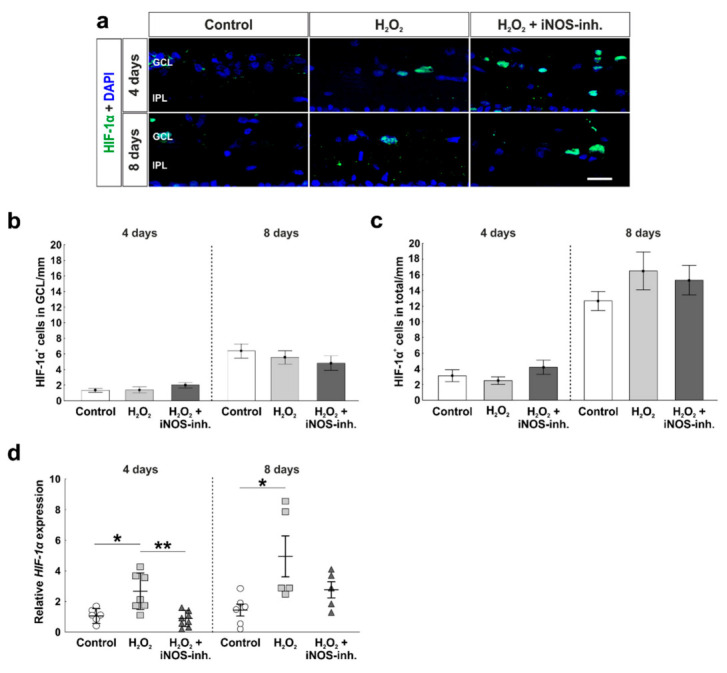
Early inhibition of *HIF-1α* gene expression. (**a**) Hypoxic cells were stained using HIF-1α antibodies (green). Cell nuclei are shown in blue. (**b**) The number of hypoxic cells was not altered in the GCL. (**c**) In the total retina, the number of HIF-1α^+^ cells was also not altered. (**d**) In contrast, *HIF-1α* mRNA expression was increased after oxidative stress at four (*p* = 0.014) and eight days (*p* = 0.02). Interestingly, the inhibition of iNOS enabled a lower expression of *HIF-1α* (four days: *p* = 0.002), more comparable to the controls. GCL = ganglion cell layer; IPL = inner plexiform layer. (**a**–**c**) n = 9–10/group and (**d**) n = 5–8/group; scale bars = 20 µm; values are shown as mean ± SEM for immunohistology and mean ± SD for RT-qPCR. * *p* < 0.01; ** *p* < 0.05.

In the present work, the effect of oxidative stress and the iNOS-inhibitor 1400 W on HIF-1α was examined (Figure 2a). At four days, no statistical differences regarding the number of HIF-1α^+^ cells in the GCL were noted within the groups (control: 1.3 ± 0.2 HIF-1α^+^ cells/mm; H_2_O_2_: 1.4 ± 0.4 HIF-1α^+^ cells/mm; *p* = 0.99; H_2_O_2_ + iNOS-inh.: 2.0 ± 0.4 HIF-1α^+^ cells/mm; *p* = 0.34). At eight days, the number of HIF-1α^+^ cells in the GCL was higher than at four days, but still comparable in all groups (control: 6.4 ± 0.9 HIF-1α^+^ cells/mm; H_2_O_2_: 5.6 ± 0.8 HIF-1α^+^ cells/mm; *p* = 0.79; H_2_O_2_ + iNOS-inh.: 4.5 ± 0.9 HIF-1α^+^ cells/mm; *p* = 0.45; Figure 2b).

Results regarding the total cell number of HIF-1α^+^ cells in the total retina matched the ones described before. Neither oxidative stress alone, nor the iNOS-inhibitor had any effects at four days (control: 3.1 ± 0.8 HIF-1α^+^ cells/mm; H_2_O_2_: 2.5 ± 0.5 HIF-1α^+^ cells/mm; *p* = 0.82; H_2_O_2_ + iNOS-inh.: 4.2 ± 0.9 HIF-1α^+^ cells/mm; *p* = 0.56). In addition, at eight days, the number of HIF-1α^+^ cells was unaltered (control: 12.7 ± 1.2 HIF-1α^+^ cells/mm; H_2_O_2_: 16.5 ± 2.4 HIF-1α^+^ cells/mm; *p* = 0.35; H_2_O_2_ + iNOS-inh.: 15.3 ± 1.9 HIF-1α^+^ cells/mm; *p* = 0.59; Figure 2c).

To investigate the hypoxic stage on the gene level, we performed RT-qPCR analysis of *HIF-1α*. Interestingly, the H_2_O_2_-induced oxidative stress led to a significantly increased expression of *HIF-1α* mRNA (2.5 ± 0.4-fold) compared to the control retinas (1.1 ± 0.2-fold; *p* = 0.014) and retinas treated with iNOS-inhibitor (0.84 ± 0.2-fold; *p* = 0.002). At eight days, *HIF-1α* mRNA expression in H_2_O_2_-stressed retinas (5.0 ± 1.4-fold) was significantly higher than in the control retinas (1.4 ± 0.4-fold; *p* = 0.02). The treatment with the iNOS-inhibitor led to a lower expression than in the H_2_O_2_-stressed retinas, but was still higher than in the controls (2.7 ± 0.5-fold; *p* = 0.49). No statistical difference was noted between the H_2_O_2_- and H_2_O_2_ + iNOS-inhibitor treated retinas (*p* = 0.2; Figure 2d).

### 3.2. Protection of Retinal Ganglion Cells and Mitochondria

**Figure 3:**
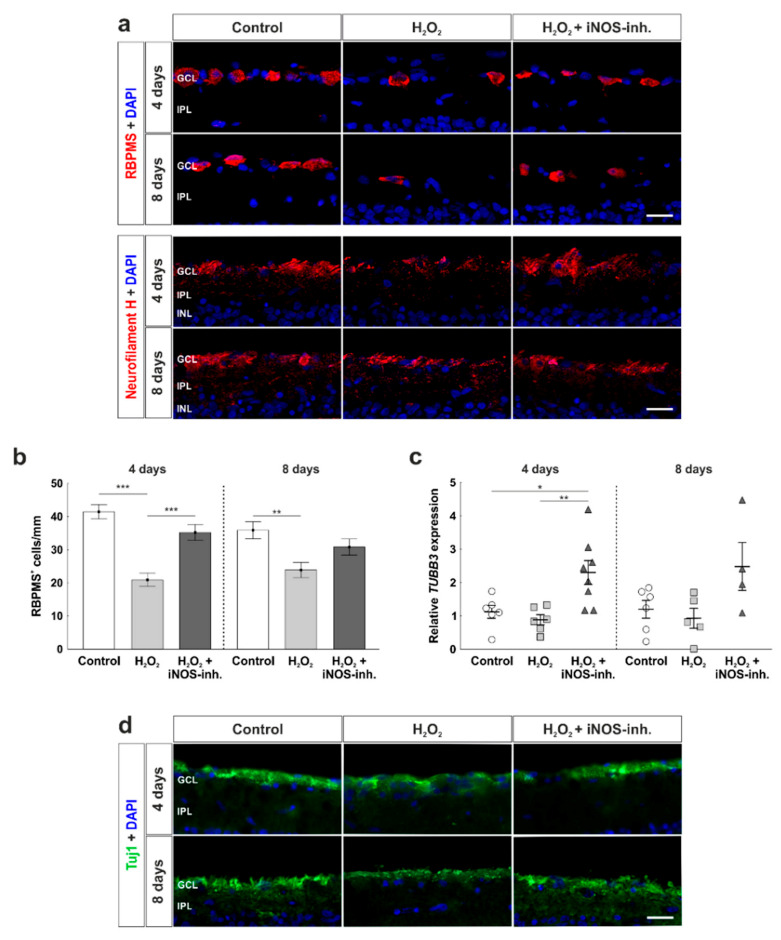

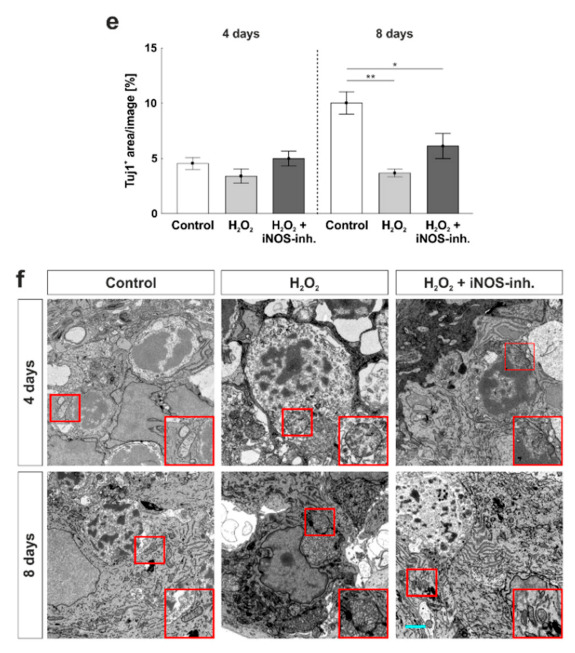
Number and morphology of retinal ganglion cells was protected. (**a**) RGCs were stained with anti-RBPMS (red, upper images) and anti-neurofilament H (red, lower images) at four and eight days. Cell nuclei were labelled with DAPI (blue). (**b**) Cell counts revealed a loss of RGCs at four (*p* < 0.001) and eight days (*p* = 0.005). This effect was strongly prevented by the iNOS-inhibitor at four days (*p* = 0.005) and attenuated at eight days (*p* = 0.13). (**c**) At four days, the iNOS-inhibitor led to a significantly increased mRNA expression of *TUBB3* (to H_2_O_2_: *p* = 0.006; to control: *p* = 0.02). At eight days, no statistical differences were seen within the groups. (**d**) Neuronal marker Tuj1 was used to investigate retinal neurons (green). Cell nuclei were labelled with DAPI (blue). (**e**) At four days, no effect of H_2_O_2_ or the iNOS-inhibitor was noted within the groups. At eight days, however, a significant smaller Tuj1^+^ area was noted in H_2_O_2_ (*p* = 0.002) as well as in the H_2_O_2_ + iNOS-inhibitor treated retinas (*p* = 0.03). Still, the Tuj1^+^ area of the H_2_O_2_ + iNOS-inhibitor treated retinas was twice as large as in H_2_O_2_ retinas. (**f**) Transmission electron microscopy revealed a preservation from oxidative stress at four and eight days. Cell morphology as well as the cell compartments of RGCs were protected by the iNOS-inhibitor. The red box indicates a mitochondrion of each group whose morphology is intact in the control and iNOS-inhibitor group and swollen in the H_2_O_2_ group GCL = ganglion cell layer; IPL = inner plexiform layer. (**a**,**b**) n = 9–10/group and (**c**) n = 6–8/group; scale bars: (**a**,**d**) 20 µm and (**f**): 5 µm; values are shown as mean ± SEM for immunohistology and mean ± SD for RT-qPCR. * *p* < 0.05; ** *p* < 0.01; *** *p* < 0.001.

To investigate the number of RGCs, RBPMS was used as a specific marker (Figure 3a). A strong loss of RGCs due to oxidative stress was seen at four days in H_2_O_2_-stressed retinas (20.9 ± 2.0 RBPMS^+^ cells/mm; *p* < 0.001) in comparison to the control group (41.5 ± 2.2 RBPMS^+^ cells/mm). Treatment with the iNOS-inhibitor led to a rescue of RGCs (35.2 ± 2.4 RBPMS^+^ cells/mm) that was so prominent that iNOS-inhibitor treated retinas had significantly more RGCs than H_2_O_2_-retinas (*p* < 0.001) and no differences were seen in comparison to the control ones (*p* = 0.15). After eight days, similar effects were noted. A strong loss of RGCs due to H_2_O_2_ (24.0 ± 2.3 RBPMS^+^ cells/mm; *p* = 0.005) in comparison to the control retinas (35.9 ± 2.6 RBMPS^+^ cells/mm) was noted. iNOS-inhibitor treated retinas (30.8 ± 2.4 RBPMS^+^ cells/mm) had more RGCs than H_2_O_2_-stressed retinas and less than the controls. However, no statistical differences were seen between iNOS-inhibitor treated and H_2_O_2_-stressed retinas (*p* = 0.14) or control ones (*p* = 0.32; Figure 3b).

Furthermore, RGCs were stained with anti-neurofilament H (Figure 3a). Neurofilament H^+^ signal was expressed in the GCL, surrounding RGCs, and also present in the intercellular space. Oxidative stress due to H_2_O_2_ seemed to weaken neurofilament H-signal compared to the control retinas. iNOS-inhibitor treated retinas had a similar staining pattern as control retinas.

RT-qPCR was performed to evaluate mRNA expression of *TUBB3* as a marker for RGCs. At four days, oxidative stress induced by H_2_O_2_ had no effect on the retinas (H_2_O_2_: 0.87 ± 0.15-fold expression; control: 1.11 ± 0.20-fold; *p* = 0.84). Interestingly, iNOS-inhibitor treated retinas (2.30 ± 0.36-fold) had a significantly increased expression of *TUBB3* in comparison to H_2_O_2_ (*p* = 0.006) as well as to the control retinas (*p* = 0.02). At eight days, *TUBB3* mRNA expression was similar in all groups (control: 1.22 ± 0.27-fold; H_2_O_2_: 0.95 ± 0.3-fold; *p* = 0.88; H_2_O_2_ + iNOS-inh.:2.54 ± 0.74-fold; *p* = 0.12). Comparing the H_2_O_2_ retinas and iNOS-inhibitor treated ones, the mRNA expression of *TUBB3* was still 2.5-fold higher (*p* = 0.065; Figure 3c).

For further analysis of retinal neurons, anti-Tuj1 was used (Figure 3d). In contrast to RBPMS-staining, no statistical differences were seen comparing all three groups at four days (control: 4.6 ± 0.5%; H_2_O_2_: 3.4 ± 0.6%; *p* = 0.42; H_2_O_2_ + iNOS-inh.: 5.0 ± 0.7%; *p* = 0.82 to control; *p* = 0.21 to H_2_O_2_). In contrast, after eight days, a significant decrease in the Tuj1^+^ area was noted in H_2_O_2_ treated retinas (3.7 ± 0.3%) in comparison to the control ones (10.0 ± 1.0%; *p* = 0.002). Even though the Tuj1^+^ area of the H_2_O_2_ + iNOS-inhibitor treated retinas was significantly smaller than in the control retinas (6.1 ± 1.1%; *p* = 0.03), the value was still twice as high as in the H_2_O_2_-damaged retinas. However, no statistical difference was seen when comparing the H_2_O_2_ and H_2_O_2_ + iNOS-inhibitor treated groups (*p* = 0.19; Figure 3e).

To evaluate the morphological effects of oxidative stress and the possible neuroprotective effects of the iNOS-inhibitor on RGCs and their cell compartments, transmission electron microscopy was performed. H_2_O_2_ had a strong impact on the morphology of RGCs at four and eight days. Disrupted retinal tissue with clearly damaged RGCs, in which cell nuclei were not as structured as in control retinas, were observed in both points in time. Furthermore, damages on cell compartments such as mitochondria were noted in H_2_O_2_-treated retinas. Morphologically, a neuroprotective effect of the iNOS-inhibitor was noted at four and eight days. Not only the tissue structure itself, but also cell compartments, like mitochondria, seemed to be protected by the treatment with the iNOS-inhibitor after four and eight days of cultivation (Figure 3f).

### 3.3. Slight Apoptosis after Oxidative Stress

**Figure 4:**
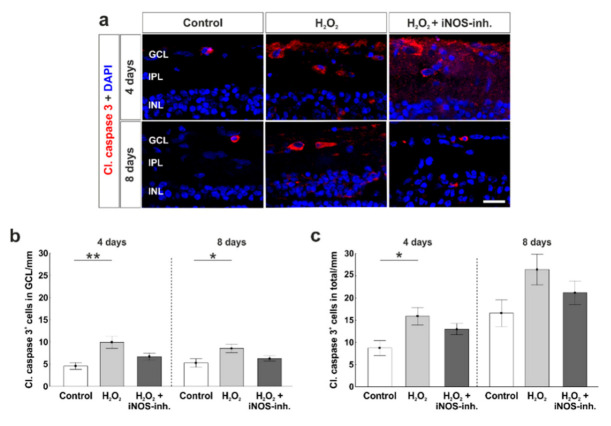

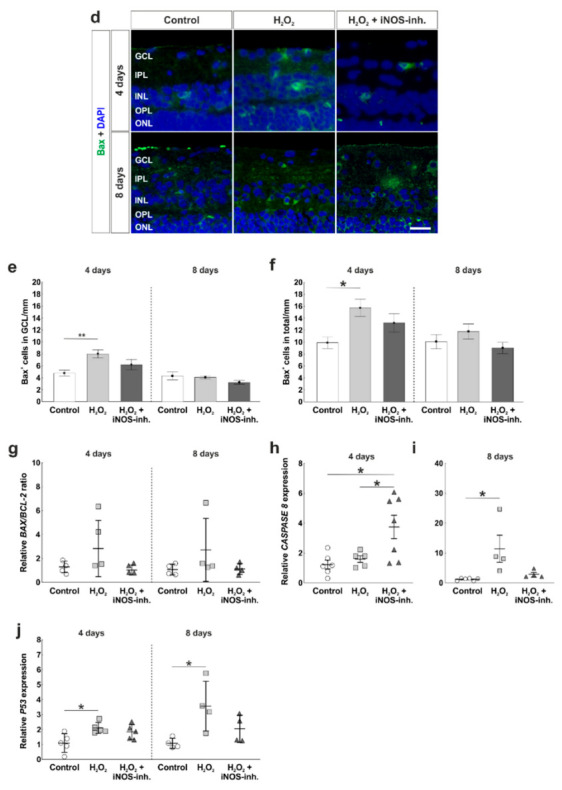
Less apoptosis in iNOS-inhibitor treated retinas. (**a**) Apoptotic cells were stained with cleaved caspase 3-antibodies (red). Cell nuclei were labelled with DAPI (blue). (**b**) A significantly increased number of apoptotic cells in the GCL was noted in H_2_O_2_-retinas at four (*p* = 0.003) and eight days (*p* = 0.03). Treatment with the iNOS-inhibitor prevented this increase. No differences were seen comparing the iNOS-inhibitor and the control group (four days: *p* = 0.32; eight days: *p* = 0.67). (**c**) Additionally, in the total retina, the number of cleaved caspase 3^+^ cells was elevated through oxidative stress (*p* = 0.015), which was prevented by the iNOS-inhibitor (*p* = 0.19). (**d**) Bax-antibodies were used to visualize apoptotic cells of the intrinsic pathway (green). Cell nuclei are shown by DAPI-staining in blue. (**e**) A higher number of Bax^+^ cells was noted in the GCL of H_2_O_2_-retinas at four days (*p* = 0.008). Treatment with the iNOS-inhibitor lowered the number of apoptotic cells, but this effect was not significant (*p* = 0.33). (**f**) In the total retina, more Bax^+^ cells were seen in H_2_O_2_-stressed retinas at four days (*p* = 0.014). The iNOS-inhibitor had no effect on the number of Bax^+^ cells (*p* = 0.21). (**g**) Comparable expressions of *BAX/BCL-2* ratio were noted in all groups. (**h**) A significantly increased mRNA expression of *CASPASE 8* was seen in iNOS-inhibitor treated groups (to control retinas: *p* = 0.015) to H_2_O_2_: *p* = 0.048). (**i**) At eight days, the expression of *CASPASE 8* mRNA due to H_2_O_2_ (*p* = 0.018) was totally counteracted by the iNOS-inhibitor. (**j**) *P53* expression was significantly increased after oxidative stress in both points in time (four days: *p* = 0.022; eight days: *p* = 0.028). The iNOS-inhibitor tended to reduce the expression of *P53* toward the control level. GCL = ganglion cell layer; IPL = inner plexiform layer; INL = inner nuclear layer; OPL = outer plexiform layer; ONL = outer nuclear layer. (**a**–**f**) n = 9–10/group and (**g**–**i**) n = 4–8/group; scale bars = 20 µm; values are shown as mean ± SEM for immunohistology and mean ± SD for RT-qPCR. * *p* < 0.05; ** *p* < 0.01.

To analyze the role of apoptotic processes during the iNOS-inhibitor treatment, we used cleaved caspase 3 antibodies to label apoptotic cells (Figure 4a). At four days, the significantly increased amount of apoptotic cells in the GCL due to H_2_O_2_ (9.9 ± 1.4 cl. caspase 3^+^ cells/mm; *p* = 0.003) in comparison to the control retinas (4.6 ± 0.76 cl. caspase 3^+^ cells/mm) was prevented by the inhibition of iNOS (6.7 ± 0.8 cl. caspase 3^+^ cells/mm; *p* = 0.084). No statistical difference was noted between the H_2_O_2_ + iNOS-inh. group and control group (*p* = 0.32) and between the H_2_O_2_ + iNOS-inh. group and the H_2_O_2_ group (*p* = 0.084). In addition, at eight days, a significantly increased number of apoptotic cells in the H_2_O_2_ group (8.6 ± 0.9 cl. caspase 3^+^ cells/mm; *p* = 0.033) was noted in comparison to controls (5.3 ± 1.0 cl. caspase 3^+^ cells/mm). Again, iNOS-inhibitor treatment lowered the number of apoptotic cells in the GCL (6.3 ± 0.6 cl. caspase 3^+^ cells/mm), where no differences in comparison to the control group (*p* = 0.67) as well as to H_2_O_2_-retinas were seen (*p* = 0.17; Figure 4b).

Cell counts in the total retina revealed similar results. Oxidative stress induced a significant increase of apoptotic cells (15.9 ± 2.0 cl. caspase 3^+^ cells/mm; *p* = 0.015) in comparison to the controls (8.7 ± 1.7 cl. caspase 3^+^ cells/mm) at four days. In addition, an inhibiting effect of the iNOS-inhibitor was seen (13.0 ± 1.3 cl. caspase 3^+^ cells/mm). Comparing H_2_O_2_ + iNOS-inhibitor treated retinas to the others, no statistical differences were observable (to control: *p* = 0.19; to H_2_O_2_: *p* = 0.45). At eight days, no impact of H_2_O_2_ and the iNOS-inhibitor was seen. All three groups had comparable numbers of caspase 3^+^ cells (control: 16.6 ± 3.0 cl. caspase 3^+^ cells/mm; H_2_O_2_: 26.4 ± 3.5 cl. caspase 3^+^ cells/mm, *p* = 0.09; H_2_O_2_ + iNOS-inh.: 21.2 ± 2.6 cl. caspase 3^+^ cells/mm, *p* = 0.55; Figure 4c).

To further analyze the intrinsic apoptotic mechanisms, antibodies against Bax, a pro-apoptotic protein, were used (Figure 4d). Counts of Bax^+^ cells in the GCL revealed a significantly increased number of apoptotic cells in H_2_O_2_-retinas (8.0 ± 0.7 Bax^+^ cells/mm; *p* = 0.008) in comparison to the control group (4.8 ± 0.5 Bax^+^ cells/mm) at four days. iNOS-inhibitor treatment reduced Bax^+^ cell number, but this effect was not significant (6.2 ± 0.9 Bax^+^ cells/mm; *p* = 0.18). At eight days, no effects were seen neither through H_2_O_2_ nor through the iNOS-inhibitor (control: 4.3 ± 0.7 Bax^+^ cells/mm; H_2_O_2_: 4.1 ± 0.2 Bax^+^ cells/mm; *p* = 0.9; H_2_O_2_ + iNOS-inh.: 3.2 ± 0.3 Bax^+^ cells/mm; *p* = 0.23; Figure 4e).

The same tendencies were seen regarding Bax^+^ cell numbers in the total retina. At four days, oxidative stress led to a significantly increased number of Bax^+^ cells (15.7 ± 1.4 Bax^+^ cells/mm; *p* = 0.014) compared to the controls (9.9 ± 0.9 Bax^+^ cells/mm). No statistical difference was notable between H_2_O_2_-stressed retinas and the iNOS-inhibitor treated group (13.2 ± 1.5 Bax^+^ cells/mm; *p* = 0.4), and counts were not that much higher than in the control group (*p* = 0.21). At eight days, the effect of H_2_O_2_ as well as the iNOS-inhibitor was not as strong. The number of Bax^+^ cells in H_2_O_2_-retinas (11.8 ± 1.3 Bax^+^ cells/mm) was not altered in comparison to the controls (10.1 ± 1.2 Bax^+^ cells/mm; *p* = 0.55). Additionally, the iNOS-inhibitor had no influence on the Bax^+^ cell number (9.0 ± 0.9 Bax^+^ cells/mm; *p* = 0.8 to control; *p* = 0.22 to H_2_O_2_; Figure 4f).

The mRNA levels of several apoptotic genes were also investigated. At four days, a 3-fold upregulation of *BAX/BCL-2* ratio was noted after H_2_O_2_-stress (3.4 ± 1.2-fold; *p* = 0.136) in comparison to the control retinas (1.2 ± 0.2-fold), although this effect was not significant. Even though no statistical difference was noted, the iNOS-inhibitor still led to a normalization of the *BAX/BCL-2* ratio compared to the H_2_O_2_-group (1.16 ± 0.2-fold; *p* = 0.12). At eight days, the *BAX/BCL-2* ratio was twice as high in the H_2_O_2_-stressed retinas (2.7 ± 1.3-fold), but no statistical difference was observed in comparison to the controls (1.1 ± 0.2-fold; *p* = 0.34). Again, retinas that received the iNOS-inhibitor treatment had comparable *BAX/BCL-2* ratio as controls (1.1 ± 0.2-fold; *p* = 0.99). Furthermore, the *BAX/BCL-2* ratio was lower than in the H_2_O_2_-retinas, although without any significance (*p* = 0.37; Figure 4g).

At four days, the analysis of *CASPASE 8* mRNA revealed no altered expression in H_2_O_2_-stressed retinas (H_2_O_2_: 1.57 ± 0.23-fold; control: 1.20 ± 0.29; *p* = 0.90). Treatment with the iNOS-inhibitor led to a significantly increased *CASPASE 8* mRNA expression (3.74 ± 0.79-fold) in comparison to the control retinas (*p* = 0.015) as well as to the H_2_O_2_ retinas (*p* = 0.048; Figure 4i). At eight days, oxidative stress increased the *CASPASE 8* expression significantly (13.45 ± 5.47-fold; *p* = 0.018) in comparison to the control retinas (1.16 ± 0.2-fold). This effect was counteracted by the iNOS-inhibitor (3.19 ± 0.77-fold), where no statistical difference was seen compared either to control (*p* = 0.85) or to H_2_O_2_-stressed retinas (*p* = 0.069; Figure 4i).

A further investigated apoptosis gene was *P53*. At four days, oxidative stress induced a significantly increased mRNA expression level of *P53* (2.1 ± 0.2-fold; *p* = 0.022) in comparison to control retinas (1.1 ± 0.3-fold). This effect was strongly reduced after iNOS-inhibitor treatment (1.8 ± 0.2-fold; *p* = 0.70), even though the *P53* expression was still higher than in the control retinas (*p* = 0.092; Figure 4j). The same effects on *P53* expression level were seen at eight days. H_2_O_2_ significantly increased the expression level of *P53* (3.6 ± 0.8-fold; *p* = 0.028) in comparison to the controls (1.1 ± 0.2-fold). Interestingly, the iNOS-inhibitor normalized the *P53* expression (2.0 ± 0.5-fold; *p* = 0.19) compared to the H_2_O_2_-retinas. In comparison to the control retinas, no statistical differences were seen anymore (*p* = 0.5; Figure 4j).

### 3.4. Early Activation of p62, an Autophagic Marker, through Oxidative Stress

**Figure 5:**
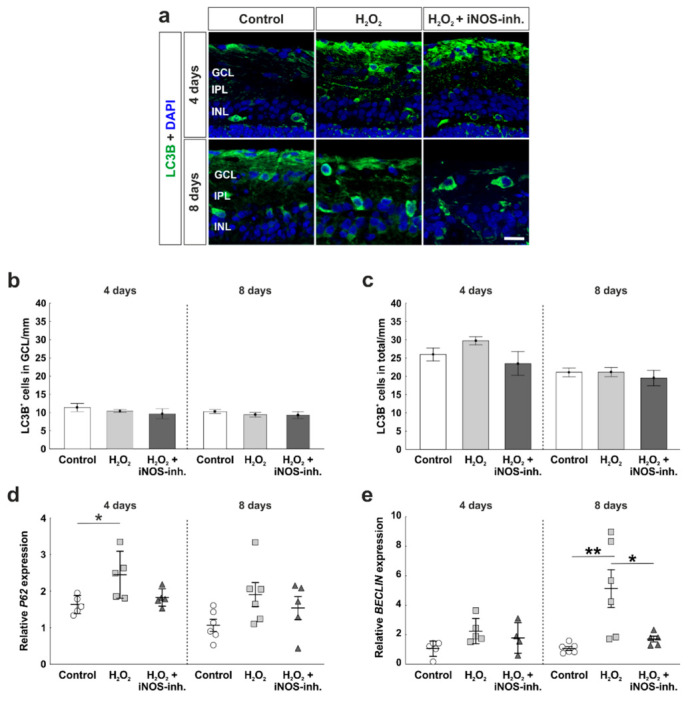
Early activation of autophagy marker. (**a**) Autophagic cells were labelled with LC3B (green) and cell nuclei are shown in blue by DAPI-staining. (**b**) No differences were seen for LC3B^+^ cells located in the GCL at four and eight days. (**c**) LC3B^+^ cell counts in the total retina were also comparable in all groups. (**d**) mRNA expression of *P62* was significantly increased through H_2_O_2_ at four days, whereas iNOS-inhibitor treated retinas had comparable *P62* expression to the controls. (**e**) No altered mRNA expression of *BECLIN* was noted after oxidative stress or iNOS-inhibitor treatment in the retinas at four days. However, at eight days, oxidative stress led to an overexpression of *BECLIN* (*p* = 0.006), this effect was counteracted by the inhibitor (*p* = 0.025). (**a**–**c**) n = 9–10/group and (**d**–**e**) n = 5–8/group; scale bar = 20 µm; values are shown as mean ± SEM for immunohistology and mean ± SD for RT-qPCR. * *p* < 0.05; ** *p* < 0.01.

A pathway that is strongly connected to apoptosis is autophagy. Possible positive effects of the iNOS-inhibitor treatment on autophagocytotic processes were analyzed immunohistochemically by using LC3B antibodies (Figure 5a). Statistical evaluation of LC3B^+^ cells located in the GCL revealed a similar number of autophagocytotic cells within all three groups at four days (control: 11.4 ± 1.1 LC3B^+^ cells/mm; H_2_O_2_: 10.4 ± 0.4 LC3B^+^ cells/mm; *p* = 0.79; H_2_O_2_ + iNOS-inh.: 9.7 ± 1.3 LC3B^+^ cells/mm; *p* = 0.47). In addition, at eight days, no statistical differences were observed (control: 10.2 ± 0.6 LC3B^+^ cells/mm; H_2_O_2_: 9.4 ± 0.7 LC3B^+^ cells/mm; *p* = 0.70; H_2_O_2_ + iNOS-inh.: 9.3 ± 0.9 LC3B^+^ cells/mm; *p* = 0.65; Figure 5b).

We further investigated the number of LC3B^+^ cells throughout the total retina. At four days, all retinas of the control (26.0 ± 1.8 LC3B^+^ cells/mm), H_2_O_2_ (29.8 ± 1.1 LC3B^+^ cells/mm; *p* = 0.47), as well as H_2_O_2_ + iNOS-inh. group (23.5 ± 3.2 LC3B^+^ cells/mm; *p* = 0.71) had similar amounts of autophagocytotic cells. In addition, at eight days, neither H_2_O_2_ nor the iNOS-inhibitor had any effects on the number of autophagocytotic cells, where the control (21.1 ± 1.2 LC3B^+^ cells/mm), the H_2_O_2_ (21.2 ± 1.3 LC3B^+^ cells/mm; *p* = 0.9), and the H_2_O_2_ + iNOS-inh. group (19.6 ± 2.1 LC3B^+^ cells/mm; *p* = 0.77) had an unaltered number of LC3B^+^ cells in the total retina (Figure 5c).

A gene, which is involved in early autophagic processes and was investigated by RT-qPCR analysis, is *P62*. Interestingly, at four days, a significantly increased mRNA expression level of *P62* was noted after oxidative stress (2.4 ± 0.5-fold; *p* = 0.025) in comparison to the control retinas (1.6 ± 0.1-fold). After the inhibition of iNOS, *P62* expression was comparable to the control retinas (1.8 ± 0.1-fold), but no statistical differences were seen compared either to H_2_O_2_ (*p* = 0.088) or to the control retinas (*p* = 0.76). At eight days, the effects of oxidative stress and the inhibitor were not as strong as at four days. Oxidative stress induced by H_2_O_2_ led to a 3.5-fold increased mRNA expression level of *P62* (3.5 ± 1.1-fold; *p* = 0.11) in comparison to the control group (1.1 ± 0.2-fold). Again, even though no statistical difference was noted, the iNOS-inhibitor treatment reduced the mRNA expression to a comparable level of the control group (1.9 ± 0.5-fold; *p* = 0.75; Figure 5d).

Another protein that is correlated with early autophagy is Beclin-1. At four days, neither H_2_O_2_ nor the iNOS-inhibitor had any effect on *BECLIN* mRNA expression (control: 1.0 ± 0.2-fold; H_2_O_2_: 2.2 ± 0.4-fold; *p* = 0.093; H_2_O_2_ + iNOS-inh.: 1.8 ± 0.5-fold; *p* = 0.43).

At eight days, H_2_O_2_-retinas had an mRNA expression level that was 5-fold higher than in the control retinas (H_2_O_2_: 5.1 ± 1.3-fold; *p* = 0.006; control: 1.0 ± 0.1-fold). The mRNA expression of *BECLIN* was significantly lowered after the iNOS-inhibitor treatment (1.7 ± 0.2-fold; *p* = 0.025). This effect was so strong that no statistical difference was noted between the iNOS-inhibitor treated retinas and control retinas (*p* = 0.84; Figure 5e).

### 3.5. Late Rescue of Rod Bipolar Cells

**Figure 6:**
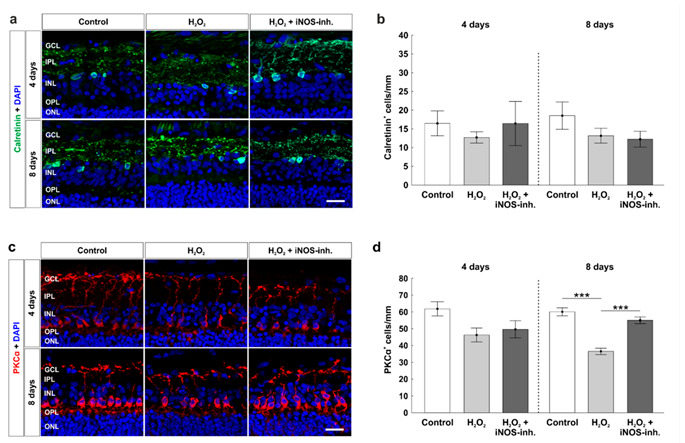
Late rescue of rod bipolar cells. (**a**) Calretinin (green) labelled amacrine cells at four and eight days. Cell nuclei were stained with DAPI (blue). (**b**) Counts of calretinin^+^ cells revealed no differences for both points in time. (**c**) Rod bipolar cells were stained with PKCα (red) at four and eight days. DAPI was used to visualize cell nuclei (blue). (**d**) At eight days, a loss of rod bipolar cells was noted in the H_2_O_2_-stressed group (*p* < 0.001). This effect was strongly diminished by the iNOS-inhibitor (*p* < 0.001). GCL = ganglion cell layer; IPL = inner plexiform layer; INL = inner nuclear layer; OPL = outer plexiform layer; ONL = outer nuclear layer. n = 9–10/group; scale bars = 20 µm; values are shown as mean ± SEM. *** *p* < 0.001.

To investigate cells of the inner nuclear layer at four and eight days, which are important for the transmission of signals from photoreceptors to RGCs, we used calretinin as a specific marker for amacrine cells (Figure 6a) and PKCα for rod bipolar cells (Figure 6c). Neither oxidative stress nor the iNOS-inhibitor treatment had any impact on the amount of calretinin^+^ amacrine cells at four days (control: 16.7 ± 3.4 calretinin^+^ cells/mm; H_2_O_2_: 12.7 ± 1.5 calretinin^+^ cells/mm; *p* = 0.75; H_2_O_2_ + iNOS-inh.: 15.9 ± 5.5 calretinin^+^ cells/mm; *p* = 0.99). At eight days, the number of calretinin^+^ cells was still similar in all groups (control: 18.5 ± 3.6 calretinin^+^ cells/mm; H_2_O_2_: 13.1 ± 2.0 calretinin^+^ cells/mm; *p* = 0.35; H_2_O_2_ + iNOS-inh.: 12.2 ± 2.1 calretinin^+^ cells/mm; *p* = 0.24; Figure 6b).

Cell counts of PKCα^+^ cells revealed a similar number in rod bipolar cells after four days in oxidative stressed retinas (45.5 ± 4.5 PKCα^+^ cells/mm, *p* = 0.07) compared to the controls (60.8 ± 4.9 PKCα^+^ cells/mm). No changes were seen after iNOS-inhibitor treatment (49.3 ± 4.6 PKCα^+^ cells/mm) compared to the control (*p* = 0.21) or H_2_O_2_-stressed retinas (*p* = 0.83). However, at eight days, a strong loss was noted in oxidative stressed retinas (36.5 ± 1.9 PKCα^+^ cells/mm; *p* < 0.001) in comparison to the control ones (60.0 ± 2.3 PKCα^+^ cells/mm). Interestingly, treatment with the iNOS-inhibitor led to a significant rescue of around 30% of rod bipolar cells (55.0 ± 2.0 PKCα^+^ cells/mm; *p* < 0.001). No changes in bipolar cells were noted when comparing the H_2_O_2_ + iNOS-inhibitor treated retinas and control ones (*p* = 0.21; Figure 6d).

## 4. Discussion

In numerous retinal diseases, such as glaucoma, the underlying degeneration processes have not yet been fully elucidated. To understand the pathological changes and to investigate possible neuroprotective approaches, reliable research models are required. Organ cultures, especially those of explanted porcine retinas, have come increasingly into focus for this. Since oxidative stress is a common feature of degenerative retina diseases, including glaucoma [19], we recently established an ex vivo degeneration model of cultured porcine retinas. There, we induced oxidative stress by adding H_2_O_2_ to the medium, which led to severe retinal damage [8]. Interestingly, in the presence of lipopolysaccharides, the expression of iNOS, and as a consequence, also the production of the radical NO in microglia, is increased by H_2_O_2_ [27]. NO has different functions as a signaling molecule and plays a crucial role in the host cell defense [13,14]. In the retina, light stimuli regulate the synthesis of NO. Moreover, it acts as a regulator of visual adjustment at various signal processing levels and modulates the light response in all retinal neurons. It activates the specific ionic conductivities in retinal cells like rods, cones, bipolar cells, and RGCs [28]. In contrast, large amounts of NO are converted into NO_2_, nitrite, peroxynitrite, and free radicals, which in turn induce pathophysiological effects such as retinal apoptosis and optic nerve degeneration. Furthermore, high concentrations of NO lead to an increased expression of *HIF-1α*, which can be toxic for cells [29]. Those pathomechanisms are involved in glaucoma [20,30]. Therefore, the inhibition of iNOS represents an interesting therapeutic approach for the treatment of neurodegenerative diseases including retinal diseases [22,31]. The iNOS-inhibitor 1400 W (N-(3-aminomethyl) benzylacetamidine) has a molecular weight of 250.17 g/mol and represents a specific irreversible iNOS-inhibitor, which suppresses the iNOS-catalyzed reaction of L-arginine to L-citrulline and thus in the end inhibits the NO production [32].

In a rat model of cerebral ischemia, the iNOS-inhibitor 1400 W reduced the infarct size and improved the neurological score [33]. The effect of the iNOS-inhibitor in further studies with primary neurons of rats and cell culture models showed that it had protecting effects by reducing apoptotic processes [34]. In a previous study, we demonstrated neuroprotective effects of the iNOS-inhibitor 1400 W on RGCs and bipolar cells in CoCl_2_-stressed hypoxic porcine retina organ cultures [5]. Now, we aimed to investigate the possible neuroprotective effects of the iNOS-inhibitor 1400 W on H_2_O_2_-damaged retinas.

First, we examined its influence on RGCs. In untreated retinas the highest number of RGCs with an intact morphological integrity were present, characterized by consistent cellular structures and intact mitochondria. Furthermore, the RGCs were organized in a uniform, dense cell cluster. In contrast, H_2_O_2_-damaged retinas had significantly fewer RGCs, which exhibited remarkable morphological changes. The oxidative stress led to uneven cell structures and an increased mitochondrial volume. In accordance, Knels et al. demonstrated that H_2_O_2_-treated retinal explants of rats displayed a structural loss of the mitochondrial intermembrane space [35]. Furthermore, ROS and oxidative stress resulted in mitochondrial damage and dysfunctions in several neurodegenerative diseases [36]. Those dysfunctions are manifested in a dysregulated oxidative phosphorylation and the release of cytotoxic factors [37]. In addition, oxidative stress leads to an altered mitochondrial membrane permeability, characterized by the abolition of the selective permeabilization of the inner mitochondrial membrane. This results in an expansion of the matrix volume [38]. With regard to our results, we also observed swollen mitochondria, which might be an expansion of the mitochondrial matrix in H_2_O_2_-stressed RGCs. This leads to the conclusion that oxidative stress induced by H_2_O_2_ led to the damage of mitochondria of the RGCs and resulted in a reduced number of RGCs. Staining of anti-Tuj1 also showed a strong loss of retinal neurons after eight days. Furthermore, we noted that Bax and cleaved caspase 3 were increased in H_2_O_2_-retinas. Hence, oxidative stress induced intrinsic apoptotic mechanisms. Increased amounts of Bax might lead through its translocation and transformation into mitochondria to mitochondrial damage and cytochrome c release, resulting in apoptosis [39], which could be observed in our study by an increased number of cleaved caspase 3^+^ RGCs.

Interestingly, treatment with 1400 W led to neuroprotective effects on RGCs. Not only was the number of RGCs almost the same in the control retinas and in the iNOS-inhibitor treated ones, but their morphology was also unaltered and the amount of Bax^+^ and cleaved caspase 3^+^ cells were reduced. Tuj1 staining did not verify these findings, which indicates that Tuj1 is not as specific to RGCs as RBPMS. In accordance, RGCs were protected against hypoxic stress through 1400 W [5]. Furthermore, the TEM results confirmed that iNOS-inhibition prevented mitochondrial changes in H_2_O_2_-stressed RGCs. The mechanism by which this protection is provided remains to be determined. The physiological and pathological roles of NO in mitochondrial function/dysfunction are still unclear. On one hand, it was described that NO can inhibit the activity of the respiratory chain complexes and on the other hand, NO is involved in the control of mitochondrial biogenesis [40].

In regard to the effect of 1400 W on apoptotic pathways, cleaved caspase 3 and Bax, as representatives of the intrinsic pathway, were analyzed. An increase in cleaved caspase 3^+^ cells in the H_2_O_2_-retinas was observed, which was lowered by the iNOS-inhibitor. In addition, the pro-apoptotic protein Bax was significantly increased in H_2_O_2_-stressed retinas at four days. This effect was alleviated by 1400 W. This is in line with Sennlaub et al., who revealed that the iNOS activity contributes to apoptotic cell death in a mouse model of ischemic proliferative retinopathy [41]. Moreover, in a rat model of renal ischemia/reperfusion, 1400 W significantly reduced the activity of caspase 3 [42].

Appropriately, oxidative stress induced by H_2_O_2_ favors the dimerization of Bax, leading to the translocation of cytosolic Bax to the outer mitochondrial membrane [36]. In addition, p53 is activated in the degenerative state of the cell, which in turn induces the transcription of genes that subsequently code for pro-apoptotic proteins such as Bax [43]. Interestingly, in our study, 1400 W prevented the H_2_O_2_-mediated increase of Bax^+^ cells. In a previous study, oxidative stress led to an increased amount of Bax [6]. The application of H_2_O_2_ leads to oxidative stress, which results in mitochondrial damage leading to the activation of the intrinsic apoptotic pathway. We suggest that 1400 W led to an increased cell survival of RGCs by inhibiting the Bax overexpression and translocation, resulting in a protection of mitochondria and therefore in a reduced intrinsic apoptosis. This is strengthened by the study of Sennlaub et al., where iNOS lacking mice as well as the iNOS-inhibitor 1400 W had positive effects by inhibiting apoptosis in a model of ischemic proliferative retinopathy [41].

Since we did not see any effect of oxidative stress on Bax^+^ cells at eight days, this might indicate an early activation of intrinsic apoptosis in our model. In further analyses of the apoptosis-signaling pathway, no differences of the mRNA expression of *CASPASE 8*, *P53*, and *BAX/BCL-2* ratio were detectable. Possibly these factors are not involved in the H_2_O_2_-mediated apoptosis. Another explanation might be that the apoptotic cascades are already in an advanced stage and that any differences are not detectable on gene level at this point in time.

Autophagy might not play such an important role in this organ culture model. Its role and function within retinal diseases continues to be discussed controversially [44]. Therefore, we examined the effect of iNOS-inhibition on autophagy via LC3B, which is of great importance in autophagosome biogenesis [45]. In addition, mRNA levels of *P62* and *BECLIN* were analyzed. *P62* is required for the aggregation of ubiquitinated proteins and their autophagic clearance and therefore used as a reporter of autophagy activity [46]. Beclin-1 is part of the initiation stage of autophagy and acts by forming the isolation membrane, which devours cytoplasmic material to form the autophagosome [47]. Whereas no effects were seen regarding LC3B^+^ cell numbers in the whole retina as well as only in the GCL, the iNOS-inhibitor led to a normalized expression of *P62* and *BECLIN*. Our results, showing that oxidative stress leads to an up-regulation of *P62*, are in line with current studies. Song et al. noted that H_2_O_2_ not only triggered the upregulation of p62 in retinal pigment epithelium (RPE) cells, but also promoted the autophagic flux in an AMD mouse model [48]. Moreover, another study using human RPE cells exhibited that p62, in response to a complex oxidant such as smoking, protects the RPE cells by reducing protein aggregates and activating a Nrf2 antioxidant response [49]. Thus, p62 provides cytoprotection against oxidative stress in retinal cells by activating the autophagic flux. In our study, likewise to the study by Song et al., an upregulation of p62 was only observed in the early point in time [48]. Interestingly, the inhibition of iNOS led to a normalized expression of *P62*.

Regarding autophagy, our results indicate that oxidative stress is only accompanied by an early upregulation of *P62* and late upregulation of *BECLIN*. This shows that autophagy does not seem to play such a crucial role in the present model, even though the inhibition of iNOS led to less *P62* expression. A crosstalk between autophagy and apoptosis could be an explanation, which leads to an interruption of the autophagic cell protective mechanism [50]. Regarding our results, we assume that autophagy is rather pro-apoptotic than protective to cells, since *P62* and *BECLIN* were only upregulated in H_2_O_2_-retinas.

It is known that hypoxic and oxidative stress can reinforce each other and the modulation of HIF-1 by NO has already been reported in various models of cultured cells [51,52]. Depending on its concentration and oxygen content, NO appears to either stabilize or destabilize HIF-1α. Therefore, we analyzed whether oxidative stress induced by H_2_O_2_ leads to an additional induction of hypoxia characterized by HIF-1α in the cultured porcine retina. In our model, on a histological level, no differences were detectable between the groups. Via RT-qPCR analyses, however, we noted that the H_2_O_2_-induced oxidative stress led to a significantly increased *HIF-1α* expression level after four days, which was prevented by 1400 W. After eight days, no statistical differences could be found, however, tendencies were similar to those at four days. Thus, 1400 W appears to be neuroprotective for the porcine retina, for instance, by inhibiting the upregulation of the hypoxic HIF-1a gene induced by H_2_O_2_. In line with those results are the one of a previous study, where the hypoxia-induced increased HIF-1α expression in a porcine retina organ culture model was significantly reduced after the inhibition of iNOS [5].

In addition to RGCs, cells of the inner retinal layer are known to be sensitive to oxidative stress. However, our analysis of the amacrine cells revealed no differences. This is in contrast with our previous study, where we detected a loss of amacrine cells after five days. However, in this previous study, the number of cholinergic amacrine cells in H_2_O_2_-stressed retinas was analyzed, whereas in the present one, all amacrine cells were investigated [6].

In contrast to the amacrine cell analysis, we detected a slightly decreased number of PKCα^+^ bipolar cells after four days and a strong cell loss after eight days in oxidative stressed retinas. Interestingly, treatment with 1400 W led to a significant rescue of the rod bipolar cells. In our previous studies, we were able to show, on one hand, that the loss of bipolar cells induced by H_2_O_2_ could also be prevented by hypothermia treatment [6] and, on the other hand, hypoxic stress also led to a loss of bipolar cells only in the late point in time, which was prevented by 1400 W [5]. Results of other studies suggest that bipolar cells represent NO sources in the inner retina and are involved in physiological and pathological processes of the inner retina via NO signal transmissions [53]. Thus, inhibition of iNOS seems to be an option to protect bipolar cells from an excessive amount of NO and thus maintain the signaling between photoreceptors and RGCs.

Therefore, a protective mechanism against oxidative stress could be observed in the porcine organ culture through treatment with the iNOS-inhibitor 1400 W. In order to get closer to a therapy option, these findings must be transferred to adequate disease animal models. One possibility to imitate the damage of the organ culture model would be to induce oxidative stress in an animal model by an intravitreal injection of a toxic substance like NMDA [26,54]. Another option would be to use the ischemia/reperfusion model, where the induced oxidative stress is accompanied by RGC loss [55]. To achieve a similar protective effect of the iNOS-inhibitor in the in vivo model, the iNOS-inhibitor could be injected intravitreally. A successful transfer would therefore be a further step toward a new therapeutic treatment option.

## 5. Conclusions

**Figure 7:**
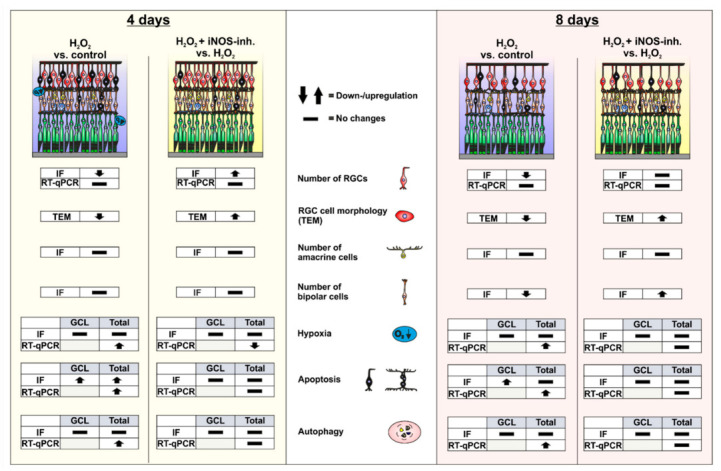
Graphical summary of the results. After four days of cultivation, morphological changes in the RGCs and loss of RGCs could be observed within H_2_O_2_-stressed retinas. In line with this, there was an increase in apoptosis in the ganglion cell layer and the total retina. In addition, an increase in the expression of hypoxic and autophagic genes could be detected. The iNOS-inhibitor 1400 W was able to stop RGC loss as well as the apoptotic, hypoxic, and autophagic processes. After eight days of cultivation, a decrease in RGC and bipolar cell numbers was observed in the H_2_O_2_-retinas. Apoptotic cells could now only be detected in the retinal ganglion cell layer. The loss of both cell types as well as some apoptotic and autophagic marker were normalized by the control by 1400 W treatment. Differences in the gene expression of hypoxic and autophagic genes could no longer be noted. GCL = ganglion cell layer; IF = immunofluorescence; TEM = transmissions electron microscopy; RT-qPCR = quantitative real-time PCR; RGC = retinal ganglion cell.

Oxidative stress is a pathomechanism that plays an important role in the development of retinal diseases. H_2_O_2_ can be used in vitro to simulate oxidative stress. In recent years, we established a screening model based on organ cultures of porcine retina. We used H_2_O_2_ to induce oxidative stress, resulting in strong neurodegenerative effects and retinal cell loss. In the present study, the possible neuroprotective effects of iNOS-inhibition in the porcine H_2_O_2_-degeneration model were investigated. Overall, H_2_O_2_ induced severe retinal degeneration after four and eight days. Oxidative stress also increased the apoptosis and autophagy. Interestingly, treatment with 1400 W prevented RGC loss to some extent. Apoptotic and hypoxic processes were somewhat alleviated. After eight days of cultivation, there was a decrease in bipolar cells in the retinas treated with H_2_O_2_, which could also be prevented by the iNOS-inhibitor (Figure 7). In summary, our results indicate that treatment with 1400 W appears to be a promising therapy option for retinal diseases.

## Figures and Tables

**Table 1:** Primary and secondary antibodies used for immunofluorescence.

Primary Antibodies					Secondary Antibodies			
Antibody	Source	Company	Research Resource Identification Number	Dilution	Antibody	Company	Research Resource Identification Number	Dilution
Anti-Bax	Rabbit	Abcam	ab7977	1:100	Donkey anti-rabbit Alexa Fluor 488	Jackson Immuno Research	711-547-003	1:500
Anti-calretinin	Goat	Merck Millipore	AB1550	1:2000	Donkey anti-goat Alexa Flour 488	Dianova	705-545-147	1:500
Anti-cleaved caspase 3	Rabbit	Sigma-Aldrich	C8487	1:300	Donkey anti-rabbit Alexa Fluor 555	Invitrogen	A31572	1:500
Anti-HIF-1α	Mouse	BD Biosciences	610959	1:100	Donkey anti-mouse Alexa Fluor 488	Abcam	A21202	1:500
Anti-LC3B	Rabbit	Cell Signaling	3868	1:300	Donkey anti-rabbit Alexa Fluor 488	Jackson Immuno Research	711-547-003	1:500
Anti-neuro-filament H	Chicken	Synaptic Systems	171106	1:300	Donkey anti-chicken Cy3	Millipore	AP194C	1:500
Anti-PKCα	Mouse	Santa Cruz	sc-8393	1:500	Donkey anti-mouse Alexa Fluor 555	Abcam	ab150106	1:500
Anti-RBPMS	Rabbit	Merck Millipore	ABN1362	1:200	Donkey anti-rabbit Alexa Fluor 555	Invitrogen	A31572	1:500
Anti-Tuj1	Mouse	Covance	MMS-435P-100	1:300	Donkey anti-mouse Alexa Flour 488	Abcam	A21202	1:500

**Table 2:** Primer pairs used in RT-qPCR analyses. Abbreviations: for = forward; rev = reverse.

Gene	Oligonucleotide Sequence (3′-5′)
*ACTB for*	CACGCCATCCTGCGTCTGGA
*ACTB rev*	AGCACCGTGTTGGCGTAGAG
*BAX for*	AAGCGCATTGGAGATGAACT
*BAX rev*	AAAGTAGAAAAGCGCGACCA
*BCL-2 for*	AATTACCATCGGCGTAGTGC
*BCL-2 rev*	CGTTTCAGCCACCGTAAAAT
*BECLIN for*	AGGAGCTGCCGTTGTACTGT
*BECLIN rev*	CACTGCCTCCTGTGTCTTCA
*CASPASE 8 for*	GCCCAGATCTCTGCCTACAG
*CASPASE 8 rev*	CAGGGCCTTGTTGATTTGTT
*HIF-1α for*	GTAATGCTCCCCTCATCCAA
*HIF-1α rev*	TGGGGCATGGTAAAAGAAAG
*P53 for*	CCTCACCATCATCACACTGG
*P53 rev*	GGCTTCTTCTTTTGCACTGG
*P62 rev*	ATGGGTCCAGTCATCGTCTC
*P62 for*	TCCAGCACAGAGGACAAGTG
*RPL4 for*	CAAGAGTAACTACAACCTTC
*RPL4 rev*	GAACTCTACGATGAATCTTC
*TUBB3 for*	CAGATGTTCGATGCCAAGAA
*TUBB3 rev*	GGGATCCACTCCACGAAGTA

## Data Availability

The datasets generated during and/or analyzed during the current study are available from the corresponding author on reasonable request.
